# Risk factors analysis and prediction model construction of perioperative gram-negative bacterial infection in patients with intestinal fistula

**DOI:** 10.1097/MD.0000000000048648

**Published:** 2026-05-08

**Authors:** Yong-Hao Sun, Kun-Jian Wei, Ming-Ming Yin, Hao-Yi Zheng, Qun Xu, Yi-Qun Shan, Ying-Nan Tian, Can-Wen Chen, Guo-Sheng Gu

**Affiliations:** a Anhui No. 2 Provincial People’s Hospital Clinical College of Anhui Medical University, Anhui No. 2 Provincial People’s Hospital, Hefei, Anhui, China; b Anhui No. 2 Provincial People’s Hospital, Hefei, Anhui, China; c Department of General Surgery, Division of Life Sciences and Medicine, The First Affiliated Hospital of USTC, University of Science and Technology of China, Hefei, Anhui, China.

**Keywords:** gram-negative bacteria, influencing factors, intestinal fistula, perioperative period, predictive model

## Abstract

The purpose of this research is to explore the factors affecting perioperative gram-negative bacterial infection in intestinal fistula patients and to construct a predictive model. Between January 2022 and June 2024, 223 individuals suffering from intestinal fistula and undergoing surgical intervention at our medical facility were chosen and allocated randomly into a training cohort (n = 156) and a validation cohort (n = 67). The determinants of perioperative gram-negative bacterial infection in these intestinal fistula patients were examined utilizing both lasso regression and logistic regression methodologies. Employing the R programming language, a linear chart model aimed at forecasting infection risk was constructed. The accuracy of this predictive model was gauged through the application of the receiver operating characteristic curve and the Hosmer–Lemeshow goodness of fit test. Furthermore, a clinical decision curve analysis was conducted to assess the practical applicability of the nomogram in a clinical context. A total of 192 gram-negative bacterial strains were identified, with 175 being multidrug-resistant organisms, accounting for 91.15%. The most prevalent was *Klebsiella pneumoniae*, with 47 strains (24.48%), followed by *Escherichia coli* at 41 strains (21.35%). Multivariate logistic regression analysis revealed that undergoing a secondary surgery during hospitalization (odds ratio [OR] = 16.97, 95% confidence interval [CI]: 1.70–168.99, *P* = .016), extended hospital stays (OR = 1.03, 95% CI: 1.01–1.05, *P* = .005), and elevated postoperative white blood cell levels (OR = 1.29, 95% CI: 1.14–1.45, *P* < .001) were independent risk factors for infection. Conversely, a higher postoperative albumin level (OR = 0.87, 95% CI: 0.80–0.94, *P* < .001) served as an independent protective factor against infection. The receiver operating characteristic curve demonstrated that the model possesses excellent discriminatory power. Additionally, the Hosmer–Lemeshow test verified the consistency of predicted and observed probabilities. Furthermore, the decision curve analysis curve indicated that the nomogram model holds significant clinical value. The nomogram model, based on whether a secondary surgery is performed, hospital stays, postoperative white blood cell count, and postoperative albumin level, exhibits high predictive performance for the occurrence of perioperative gram-negative bacterial infection in patients with intestinal fistula. This model holds significant importance for clinical prediction, guiding antibiotic usage, and postoperative care.

## 1. Introduction

Intestinal fistula is a pathological passage connecting the digestive tract with the abdominal cavity, body surface, or other organs. It can be categorized into intraluminal fistula, intestinal-skin fistula, and intestinal air fistula.^[1]^ Intestinal fistula can cause the contents of the digestive tract to flow into other organs, chambers or outside the body, resulting in infection, fluid loss, acid–base balance, water and electrolyte disorders, multiple organ dysfunction, and even death. Numerous factors can contribute to the development of intestinal fistula, with surgery being the primary cause, often occurring as a complication following gastrointestinal surgery. Tumors can also cause intestinal fistula, which is usually seen in patients with advanced gastrointestinal tumors. Fistula can be formed due to the rupture of the gastrointestinal wall caused by tumor invasion, or intestinal fistula can be caused after tumor surgery or radiotherapy. Additionally, inflammatory bowel disease, intestinal obstruction, acute pancreatitis, and certain medications are also recognized causes of intestinal fistula.^[2,3]^ A retrospective study involving 3078 patients with intestinal fistula showed that although the cure rate of intestinal fistula reached 89%, the mortality rate was still 8.5%.^[4]^ If patients with intestinal fistula were also complicated with severe abdominal infection, the mortality rate would be close to 30%.^[5]^ Furthermore, a 1-year national multi-center study involving 1521 patients with intestinal fistula indicated a mortality rate of 7.4%. In patients with intestinal fistula, complications like sepsis, multiple organ dysfunction syndrome, and massive bleeding were recognized as independent predictors of death.^[6]^

With the advancement of research, infection has emerged as a vital factor in forecasting the outcomes for individuals suffering from intestinal fistula. Clinically, gram-negative bacteria are the primary pathogens responsible for infections in intestinal fistula patients. A study on the bacteriological trends and bacterial resistance among patients with gastrointestinal fistula in tertiary hospitals in China revealed that 47.2% of intestinal fistula patients were concurrently infected with multiple microbial strains. Among the isolated bacteria, gram-negative bacteria accounted for 73.0%, with *Escherichia coli* making up 24.2%, followed by *Klebsiella pneumoniae* at 14.1%.^[5]^ Other pathogens included Enterococcus (10%), *Acinetobacter baumannii* (9%), *Pseudomonas aeruginosa* (8%), and others. Moreover, the extended application of broad-spectrum antibiotics has led to antibiotic resistance in a variety of bacteria, thereby making the treatment of intestinal fistula patients more complex.

Currently, the treatment methods for intestinal fistula are gradually being refined through clinical exploration, and the overall treatment can be divided into 3 stages^[7]^: early anti-infection and resuscitation stage, non-surgical treatment stage, and definitive surgical stage. During the treatment process, it is essential to identify the location of the fistula and the main problems faced by the patient at the current stage, and develop a targeted individualized treatment plan. Although with the support of technologies such as double-lumen cannulas negative pressure drainage devices, fibrin glue to seal fistula openings, endoscopic anastomotic clips, and enteral and parenteral nutrition, most patients with intestinal fistula can be cured through non-surgical methods. However, there are still many patients whose fistula cannot heal spontaneously. When patients meet the conditions of improved nutritional status, abdominal adhesion resolution, and organ function recovery, definitive surgical treatment can be implemented.^[8]^ Clinically, doctors will develop an individualized surgical plan for fistula excision and digestive tract reconstruction based on various conditions such as the degree of abdominal adhesion, the location of the fistula, and abdominal wall defects.^[9]^ Moreover, during the conservative treatment of intestinal fistula patients, if severe abdominal infection, gastrointestinal bleeding, etc occur, emergency surgical intervention is also required. Due to the long-term risks of electrolyte imbalance, acid–base disorders, and malnutrition in patients with intestinal fistula, surgery for these patients is more complex compared to other gastrointestinal surgeries, and the risk of postoperative complications, especially infection, is higher, seriously affecting the prognosis of patients.

Consequently, investigating perioperative infections in individuals with intestinal fistulas holds great significance. At present, limited research has been conducted on the risk factors associated with gram-negative bacterial infections in these patients during the perioperative phase. Our study is designed to delve into the etiological traits and risk factors of gram-negative bacterial infections in intestinal fistula patients in the perioperative period, and to develop a relevant predictive model. The objective is to lower the rate of perioperative infections in such patients and offer scientific basis and guidance for clinical applications.

## 2. Study subjects and methods

### 2.1. Study subjects

A retrospective analysis was conducted on the clinical data of 223 patients with intestinal fistula who underwent surgical treatment at the Second People’s Hospital of Anhui Province from January 2022 to June 2024. All patients were diagnosed with intestinal fistula through angiography and imaging examinations. Inclusion criteria: definite diagnosis of intestinal fistula, including small intestinal fistula, duodenal fistula, large intestinal fistula, gastric fistula, and large-small intestinal fistula, among others; surgery performed during hospitalization. Exclusion criteria: patients with intestinal fistula who did not undergo surgical treatment; patients younger than 18 years old; patients who died or were discharged within 24 hours after surgery; patients with only gram-positive bacterial or fungi infection during hospitalization; patients with incomplete medical records. The ethics committee of the Second People’s Hospital of Anhui Province granted approval for this study, assigning it the ethics code (R) 2024–112.

**Figure 1.**
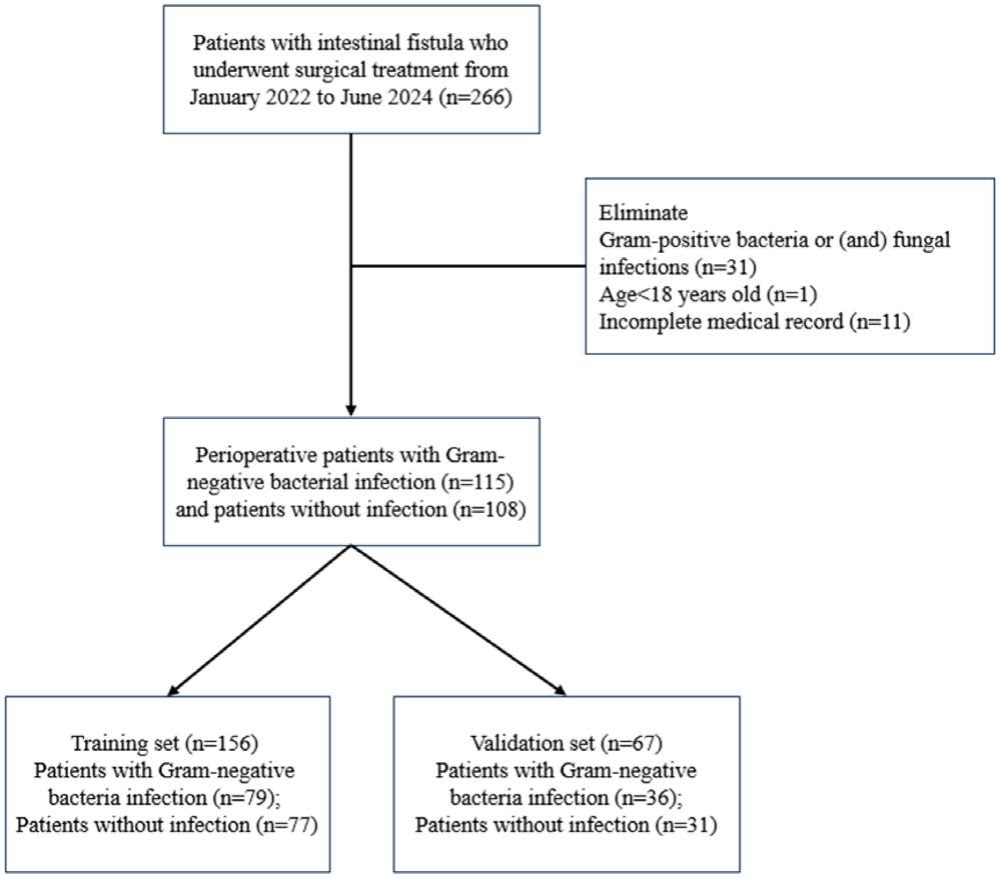
Flow chart of patients enrolled in the second people’s Hospital of Anhui Province from January 2021 to June 2024.

Initially, we identified 266 patients with intestinal fistula who underwent surgical treatment. After excluding patients with perioperative infection of gram-positive bacteria or fungi (n = 31), age <18 years (n = 1), and incomplete medical records (n = 11), the remaining 223 patients (infected with gram-negative bacteria n = 115, uninfected n = 108) were included in the final analysis. Then, the 223 patients were randomly divided into training set (n = 156, infected with gram-negative bacteria n = 79, uninfected n = 77) and validation set (n = 67, infected with gram-negative bacteria n = 36, uninfected n = 31) according to the ratio of 7:3 to construct the prediction model (Fig. 1).

### 2.2. Data collection

Based on the occurrence of gram-negative bacterial infection during the perioperative period among 223 patients with intestinal fistula, they were categorized into an infection group (n = 115) and a non-infection group (n = 108). The infection group comprised patients who developed gram-negative bacterial infection during the perioperative period, with specific diagnostic criteria requiring the simultaneous fulfillment of the following conditions: microbiological evidence: isolation of gram-negative bacteria from blood, abdominal drainage fluid, surgical incision secretions, or specimens obtained from sterile sites during surgery; clinical symptoms: presence of at least 1 infection-related sign, including body temperature exceeding 38.5°C, local erythema and edema, purulent drainage, aggravated abdominal pain, or manifestations of systemic inflammatory response syndrome; laboratory inflammatory markers: serum procalcitonin level ≥0.5 ng/mL or C-reactive protein (CRP) level ≥50 mg/L, with these abnormalities not attributable to other causes (e.g., noninfectious inflammation, transfusion reaction). Among these criteria, microbiological evidence was mandatory, while at least one of the clinical symptoms or inflammatory markers had to be present. Patients in the non-infection group did not meet any of the aforementioned criteria and had no other clinically diagnosed infections at other sites or of other types during the perioperative period.

Data were collected from both patient groups, encompassing gender, age, type of intestinal fistula, cause of intestinal fistula, type of operation (emergency/elective), operation duration, intraoperative blood loss, number of postoperative double-lumen negative pressure drainage devices, total length of hospital stay, length of ICU stay, whether there was a 2nd surgery (2 operations performed during the same hospitalization), and comorbidities (diabetes, hypertension, and malignant tumor). Clinical information includes the most recent preoperative and 1st postoperative laboratory test indicators. Microbiological data comprised sample type, bacterial species, and multidrug resistance status. Multidrug-resistant organisms were defined as pathogens exhibiting acquired resistance to at least 3 classes of commonly used antibiotics. The commonly used antibiotic classes included: anti-pseudomonal penicillins (e.g., piperacillin/tazobactam, ticarcillin/clavulanic acid); 3rd- and 4th-generation cephalosporins (e.g., ceftazidime, cefepime); carbapenems (e.g., meropenem, imipenem, ertapenem); aminoglycosides (e.g., gentamicin, amikacin, tobramycin); fluoroquinolones (e.g., ciprofloxacin, levofloxacin); sulfonamides (e.g., cotrimoxazole); and monobactams (e.g., aztreonam). Drug resistance was determined in strict accordance with the latest versions of the antimicrobial susceptibility testing standards issued by the Clinical and Laboratory Standards Institute or the European Committee on Antimicrobial Susceptibility Testing, utilizing disk diffusion, broth microdilution, or automated antimicrobial susceptibility testing systems for detection.

### 2.3. Statistical analysis

Data processing was carried out using the SPSS 26.0 statistical software and the R programming language. For measurement data, normality tests were performed. Data conforming to a normal distribution were characterized by the mean ± standard deviation (X ± s). Comparisons between the 2 groups were made using independent sample *t*-tests. Categorical data were presented as percentages (%) and inter-group comparisons were conducted via the χ^2^ test. Initially, Lasso regression was applied for variable selection. Following this, both univariate and multivariate logistic regression analyses were performed on the selected variables to pinpoint independent risk factors. A predictive model was then developed based on the findings of the multivariate logistic regression. In the R language, the rms package was utilized to create nomograms, receiver operating characteristic (ROC) curves, and calibration curves. The clinical utility of the model at various threshold levels was assessed through decision curve analysis (DCA). A *P*-value of <.05 was deemed to indicate statistical significance.

## 3. Results

### 3.1. Clinical features of patients in the infected and non-infected cohorts

The research involved 223 hospitalized patients who met the criteria and underwent surgery. Of these, 115 patients (51.57%) developed gram-negative bacterial infections during the perioperative period, whereas the remaining 108 patients (48.43%) did not experience such infections. The proportion of small intestinal fistula (infected cohort [41.74%], non-infected cohort [50.93%]) and anastomotic fistula (infected cohort [39.13%], non-infected cohort [23.15%]) is the highest in both cohorts of patients. In terms of age, there were significant differences between the infected cohort and the non- infected cohort (57.00 [47.00–68.00] vs 53.00 [42.50–63.25], *P* < .001). No significant differences were observed in gender, comorbidity prevalence, administration of radiotherapy/chemotherapy, or surgical duration between the 2 cohorts (Table 1). However, the infected cohort had a higher proportion of emergency surgeries (36.52% vs 16.67%, *P* < .001), greater intraoperative blood loss (100.00 [50.00–200.00] vs 50.00 [20.00–100.00], *P* < .001), more postoperative double-lumen cannulas (1.00 [1.00–2.00] vs 1.00 [0.00–1.00], *P* < .001), and a higher rate of secondary surgeries (22.61 vs 0.93%, *P* < .001). Patients in the infected cohort also had longer hospital stays (4.00 [2.00–10.00] vs 2.00 [1.00–3.00], *P* < .001) and ICU stays (50.00 [30.00–72.50] vs 35.00 [27.00–49.00], *P* < .001). Table 1 shows the laboratory results. There were significant differences in some preoperative and postoperative indicators (such as white blood cell [WBC] count, lymphocyte count, hemoglobin level, etc) between the infection cohort and the non-infected cohort (*P* < .001). In addition, the mortality rate of infected patients was significantly higher than that of non-infected patients (31.30 vs 1.85%, *P* < .001) as detailed in Table 1.

**Table 1:** Clinical characteristics of patients in the infected and non-infected cohorts.

Variables	Infected cohort (n = 115)	Non-infected cohort (n = 108)	*P*
Age (yr)	57.00 (47.00–68.00)	53.00 (42.50–63.25)	<.001*
Gender, n (%)			
Male	54 (50.00)	66 (57.39)	.269
Female	54 (50.00)	49 (42.61)	
Categories of intestinal fistula, n (%)			
Small intestinal fistula	48 (41.74)	55 (50.93)	
Colon fistula	31 (26.96)	26 (24.07)	
Small intestinal–colon fistula	4 (3.48)	5 (4.63)	
Duodenal fistula	1 (0.93)	13 (11.30)	
Gastric fistula	4 (3.48)	3 (2.78)	
Others	15 (13.04)	18 (16.67)	
Causes of intestinal fistula, n (%)			
Anastomotic fistula	45 (39.13)	25 (23.15)	
Gastrointestinal perforation	28 (24.35)	22 (20.37)	
Tumor invasion	17 (14.78)	10 (9.26)	
Radiation injury	10 (8.70)	15 (13.89)	
Crohn disease	3 (2.61)	12 (11.11)	
Abdominal injury	4 (3.48)	5 (4.63)	
Others	8 (6.96)	19 (17.59)	
Comorbidities, n (%)	68 (59.13)	59 (54.63)	.498
Hypertension	12 (10.43)	13 (12.04)	.705
Diabetes	9 (7.83)	8 (7.41)	.906
Malignant tumors	60 (52.17)	51 (47.22)	.460
Radiotherapy or chemotherapy, n (%)	36 (31.30)	36 (33.33)	.746
Surgical duration (min)	198.00 (132.00–270.00)	172.50 (134.50–215.00)	.161
Surgical type, n (%)			
Emergency operation	42 (36.52)	18 (16.67)	<.001*
Selective operation	73 (63.48)	90 (83.33)	
Intraoperative blood loss (mL)	100.00 (50.00–200.00)	50.00 (20.00–100.00)	<.001*
Number of postoperative double cannula	1.00 (1.00–2.00)	1.00 (0.00–1.00)	<.001*
Secondary surgery	26 (22.61)	1 (0.93)	<.001*
ICU stays (d)	4.00 (2.00–10.00)	2.00 (1.00–3.00)	<.001*
Hospital stays (d)	50.00 (30.00–72.50)	35.00 (27.00–49.00)	<.001*
Variables	Infected cohorts (n = 115)	Non-infected cohorts (n = 108)	*P*
Preoperative laboratory examination			
WBC (×10^9^/L)	6.26 (5.06–9.38)	5.82 (4.51–8.12)	.061
N (×10^9^/L)	5.15 (3.44–7.99)	3.72 (2.77–5.74)	.001*
L (×10^9^/L)	0.86 (0.55–1.23)	1.25 (0.83–1.68)	<.001*
Hb (×10^9^/L)	94.00 (82.50–111.00)	112.00 (98.75–124.25)	<.001*
PLT (×10^9^/L)	186.00 (138.50–255.00)	200.00 (166.00–261.50)	.095
ALB (g/L)	33.40 (29.40–37.00)	36.55 (32.90–40.15)	<.001*
CRP (mg/L)	42.20 (20.15–88.80)	23.35 (8.10–39.65)	<.001*
TBIL (μmol/L)	15.40 (9.60–26.25)	9.85 (6.75–14.77)	<.001*
ALT (U/L)	25.00 (14.00–42.00)	23.50 (16.75–39.25)	.747
Cr (μmol/L)	59.00 (46.00–80.50)	59.00 (48.00–70.00)	.819
D-dimer (mg/L)	1.40 (0.77–3.19)	0.67 (0.44–1.04)	<.001*
Postoperative laboratory examination			
WBC (×10^9^/L)	13.00 (8.46–17.37)	6.72 (5.28–8.92)	<.001*
N (×10^9^/L)	11.29 (7.52–15.61)	5.12 (3.69–7.11)	<.001*
L (×10^9^/L)	0.70 (0.45–1.15)	1.00 (0.69–1.37)	.001
Hb (×10^9^/L)	90.04 ± 18.53	99.63 ± 14.35	<.001*
PLT (×10^9^/L)	175.00 (108.00–271.50)	220.00 (172.25–291.50)	.002
ALB (g/L)	30.70 ± 6.11	35.96 ± 4.23	<.001*
CRP (mg/L)	78.60 (47.05–193.15)	43.10 (23.08–67.80)	<.001*
TBIL (μmol/L)	22.10 (15.00–40.95)	12.30 (7.90–19.90)	<.001*
ALT (U/L)	23.00 (16.50–39.00)	23.00 (13.75–45.75)	.874
Cr (μmol/L)	58.00 (43.50–83.50)	50.00 (40.00–66.00)	.010*
D-dimer (mg/L)	2.66 (1.66–5.22)	2.36 (1.77–3.40)	.084
Death, n (%)	36 (31.30)	2 (1.85)	<.001*

ALB = albumin, ALT = alanine aminotransferase, Cr = creatinine, CRP = C-reactive protein, Hb = hemoglobin, L = lymphocyte, N = neutrophils, PLT = platelet, TBIL = total bilirubin, WBC = white blood cell.

* *P* < .05.

### 3.2. Analysis of pathogenic characteristics of gram-negative bacteria

**Figure 2.**
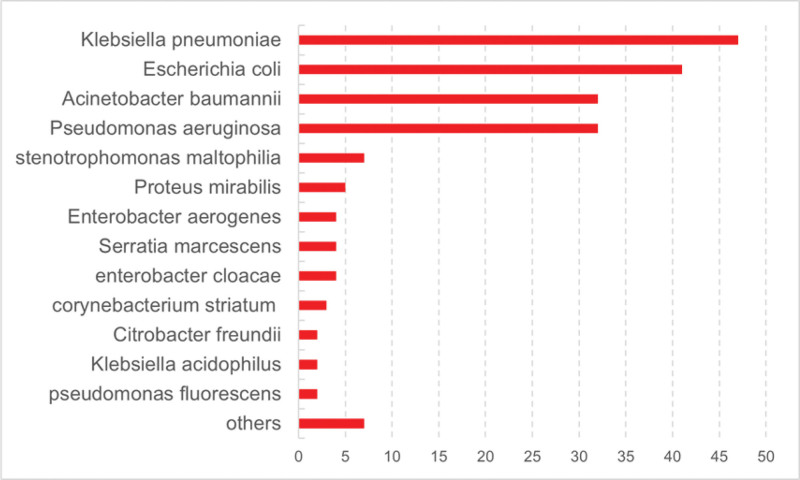
Species and distribution of gram-negative bacteria.

**Figure 3.**
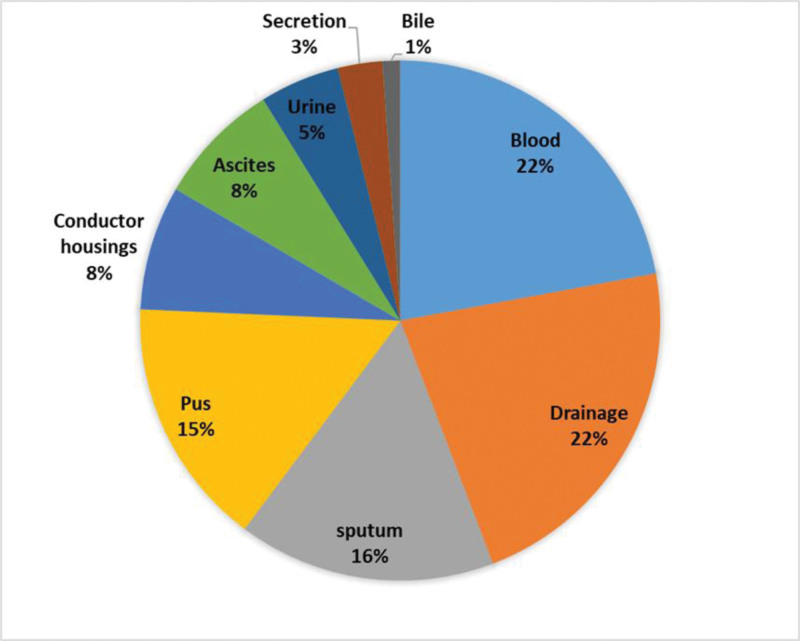
Sources of gram-negative bacteria.

A total of 192 strains of gram-negative bacteria were isolated from the infection group cohort. Out of these, 175 strains (91.15%) were identified as multidrug-resistant bacteria, while 17 strains (8.85%) were not multidrug-resistant. Overall, *K pneumoniae* emerged as the most commonly detected bacterial species, comprising 47 strains and making up 24.48% of the gram-negative isolates. *E coli* ranked 2nd, constituting 41 strains and 21.35% of the gram-negative isolates, with further details presented in Figure 2 and Table 2. The origins of the gram-negative bacteria are depicted in Figure 3, primarily consisting of blood (22%), drainage (22%), sputum (16%), pus (15%), conductor housings (8%), and ascites (8%).

**Table 2:** Distribution of gram-negative bacteria.

Pathogenic bacteria	Number of strains	Ratio (%)
*Klebsiella pneumoniae*	47	24.48%
*Escherichia coli*	41	21.35%
*Acinetobacter baumannii*	32	16.67%
*Pseudomonas aeruginosa*	32	16.67%
*Stenotrophomonas maltophilia*	7	3.65%
*Proteus mirabilis*	5	2.60%
*Enterobacter aerogenes*	4	2.08%
*Serratia marcescens*	4	2.08%
*Enterobacter cloacae*	4	2.08%
*Corynebacterium striatum*	3	1.56%
*Citrobacter freundii*	2	1.04%
*Klebsiella acidophilus*	2	1.04%
*Pseudomonas fluorescens*	2	1.04%
Others	7	3.65%
Total	192	

### 3.3. Equilibrium test of training set and verification set

This study encompassed a total of 223 eligible inpatients. The determination of the overall sample size adhered to the sample size estimation principles for constructing predictive models, employing the events per variable (EPV) criterion. Specifically, the infection group comprised 115 cases, yielding an EPV of approximately 11.5, which satisfies the widely accepted threshold of ≥10 EPV and falls within an acceptable range for predictive modeling research. The sample size was constrained by the actual capacity for enrolling patients undergoing intestinal fistula surgery at a single center. The total sample was randomly allocated into a training set (n = 156) and a validation set (n = 67) at a ratio of 7:3, a practice consistent with the conventional data division ratios employed in predictive model research (typically 70%/30% or 80%/20%). Although the validation set in this study, consisting of 67 cases, did not attain the ideal sample size for independent external validation, 3 key points warrant clarification: firstly, the validation set in this study was derived from an internal random split, primarily aimed at conducting a preliminary internal validation of model performance rather than achieving a fully powered independent external validation; secondly, the sample size of the validation set was dictated by the total sample size and the split ratio, encompassing approximately 35 infection events and 32 non-infection events within the 67-case validation set, which suffices for a preliminary assessment of model discrimination; thirdly, as delineated in Table 3, no statistically significant differences were observed in various indicators between the training set and the validation set, indicating a well-balanced random split and lending a certain degree of credibility to the validation results.

**Table 3:** Equilibrium test of training set and verification set.

Variables	Training set (n = 156)	Validation set (n = 67)	*P*
Age (yr)	57.00 (47.00–68.25)	59.00 (49.50–67.50)	.161
Gender, n (%)			
Male	86 (55.13)	34 (50.75)	.547
Female	70 (44.87)	33 (49.25)	
Infection or not, n (%)			
Infection	79 (50.64)	36 (53.73)	.672
no infection	77 (49.36)	31 (46.27)	
Comorbidities, n (%)	88 (56.41)	39 (58.21)	.804
Hypertension	16 (10.26)	9 (13.43)	.491
Diabetes	11 (7.05)	6 (8.96)	.623
Malignant tumors	78 (50.00)	33 (49.25)	.919
Radiotherapy or chemotherapy, n (%)	52 (33.33)	20 (29.85)	.610
Surgical duration (min)	183.00 (135.00–235.25)	171.00 (127.00–230.00)	.612
Surgical type, n (%)			
Emergency operation	41 (26.28)	19 (28.36)	.749
Selective operation	115 (73.72)	48 (71.64)	
Intraoperative blood loss (mL)	50.00 (20.00–100.00)	50.00 (20.00–200.00)	.904
Number of postoperative double cannula	1.00 (0.00–2.00)	1.00 (0.00–2.00)	.515
Secondary surgery	19 (12.18)	8 (11.94)	.960
ICU stays (d)	3.00 (2.00–5.00)	2.00 (1.00–4.50)	.376
Hospital stays (d)	41.00 (29.00–56.25)	46.00 (26.50–70.50)	.522
Preoperative laboratory examination			
WBC (×10^9^/L)	5.96 (4.69–8.78)	6.23 (4.82–8.54)	.985
N (×10^9^/L)	4.13 (2.86–6.99)	5.03 (3.34–6.46)	.437
L (×10^9^/L)	1.07 (0.66–1.53)	0.91 (0.60–1.26)	.086
Hb (×10^9^/L)	105.00 (89.00–122.00)	99.00 (88.00–111.00)	.093
PLT (×10^9^/L)	196.00 (147.75–260.25)	191.00 (152.00–258.00)	.717
ALB (g/L)	34.95 (31.00–38.92)	34.40 (31.65–38.80)	.522
CRP (mg/L)	29.05 (14.03–57.03)	31.20 (13.60–79.30)	.611
TBIL (μmol/L)	12.35 (7.97–20.42)	12.60 (7.40–20.25)	.852
ALT (U/L)	24.00 (15.00–42.00)	25.00 (17.50–37.00)	.622
Cr (μmol/L)	57.00 (47.00–76.25)	59.00 (47.00–77.50)	.579
D-dimer (mg/L)	0.87 (0.55–2.04)	0.95 (0.57–2.00)	.921
Variables	Training set (n = 156)	Validation set (n = 67)	*P*
Postoperative laboratory examination			
WBC (×10^9^/L)	8.66 (6.29–13.69)	9.58 (5.56–13.05)	.832
N (×10^9^/L)	7.17 (4.57–11.62)	7.84 (4.27–11.75)	.824
L (×10^9^/L)	0.86 (0.52–1.30)	0.81 (0.53–1.20)	.603
Hb (×10^9^/L)	95.75 ± 17.05	92.21 ± 17.67	.161
PLT (×10^9^/L)	200.00 (145.75–280.75)	201.00 (133.50–285.50)	.903
ALB (g/L)	33.26 ± 6.22	33.22 ± 5.09	.958
CRP (mg/L)	59.65 (30.00–96.95)	59.40 (33.65–113.61)	.988
TBIL (μmol/L)	17.35 (10.95–27.02)	14.30 (10.20–26.55)	.534
ALT (U/L)	23.00 (14.00–41.00)	23.00 (17.00–40.50)	.855
Cr (μmol/L)	53.00 (41.00–70.25)	54.00 (43.50–75.00)	.440
D-dimer (mg/L)	2.44 (1.72–4.16)	2.52 (1.65–4.21)	.931
Death, n (%)	29 (18.59)	9 (13.43)	.348

ALB = albumin, ALT = alanine aminotransferase, Cr = creatinine, CRP = C-reactive protein, Hb = hemoglobin, L = lymphocyte, N = neutrophils, PLT = platelet, TBIL = total bilirubin, WBC = white blood cell.

### 3.4. Lasso regression was employed to select characteristic factors in the training set

**Figure 4.**
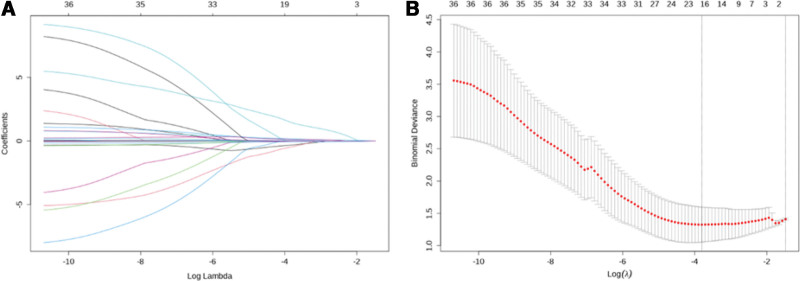
Lasso regression analysis in training set. (A) Ten times cross-validation was used to make a vertical line at the selected value, the ideal λ generates 17 non-zero coefficients. (B) In lasso model, the coefficient distributions of 36 texture features are plotted based on log (λ) sequence. The minimum mean square error (λ = 1.089) and the standard error of the minimum distance (λ = 0.081) are represented by vertical lines.

Lasso regression analysis was performed on the independent variables (Fig. 4). Lasso regression has the ability to compress variable coefficients, thereby mitigating overfitting and addressing significant multicollinearity issues. The analysis revealed that the number of independent variables was reduced from 36 to 17, including age, intraoperative blood loss, preoperative WBC, preoperative CRP, preoperative alt, preoperative creatinine, preoperative D-dimer, postoperative WBC, postoperative L, postoperative hemoglobin, postoperative platelet, postoperative albumin (ALB), postoperative CRP, length of hospital stays, secondary surgery, the number of postoperative double catheters and diabetes.

### 3.5. Univariate and multivariate logistic regression analysis

Subsequently, we explored the risk factors associated with perioperative gram-negative bacterial infections in patients with intestinal fistula. As detailed in Table 4, to further account for potential confounding factors, we performed univariate and multivariate logistic regression analyses on the 17 independent variables that were selected. Ultimately, we identified that secondary surgery (odds ratio [OR] = 16.97, *P* = .016), prolonged hospital stays (OR = 1.03, *P* = .005), and elevated postoperative WBC levels (OR = 1.29, *P* < .001) were independent risk factors for gram-negative bacterial infection. Increased postoperative ALB levels (OR = 0.87, *P* < .001) emerged as an independent protective factor against infection (Table 4).

**Table 4:** Univariate and multivariate regression analysis of factors related to perioperative gram-negative bacterial infection in patients with intestinal fistula in the training set.

Variables	Univariate analysis			Multivariate analysis	
	OR (95% CI)	*P*	OR (95% CI)		*P*
Age (yr)	1.03 (1.01–1.06)	.003*			
Intraoperative blood loss (mL)	1.01 (1.01–1.01)	.011*			
Number of postoperative double-lumen cannulas	1.57 (1.13–2.18)	.007*			
Secondary surgery	22.43 (2.91–172.75)	.003*	16.97 (1.70–168.99)		.016*
Hospital stays (d)	1.03 (1.01–1.04)	.001*	1.03 (1.01–1.05)		.005*
Preoperative WBC (×10^9^/L)	1.07 (0.99–1.15)	.084			
Preoperative CRP (×10^9^/L)	1.01 (1.01–1.01)	.020*			
Preoperative ALT (×10^9^/L)	1.00 (0.99–1.01)	.500			
Preoperative Cr (g/L)	1.00 (1.00–1.01)	.419			
Preoperative D-dimer (mg/L)	1.29 (1.09–1.53)	.004*			
Postoperative WBC (×10^9^/L)	1.31 (1.18–1.44)	<.001*	1.36 (1.14–1.63)		<.001*
Postoperative L (×10^9^/L)	0.68 (0.40–1.16)	.158			
Postoperative Hb (×10^9^/L)	0.97 (0.95–0.99)	.001*			
Postoperative PLT (×10^9^/L)	1.00 (0.99–1.00)	.088			
Postoperative ALB (g/L)	0.82 (0.76–0.88)	<.001*	0.87 (0.80–0.94)		<.001*
Postoperative CRP (mg/L)	1.01 (1.01–1.01)	.002*			
Diabetes	2.78 (0.71–10.90)	.143			

ALB = albumin, ALT = alanine aminotransferase, CI = confidence interval, Cr = creatinine, CRP = C-reactive protein, Hb = hemoglobin, L = lymphocyte, N = neutrophils, OR = odds ratio, PLT = platelet, TBIL = total bilirubin, WBC = white blood cell.

* *P* < .05.

### 3.6. Construction of predictive model for perioperative gram-negative bacterial infection in patients with intestinal fistula

**Figure 5.**
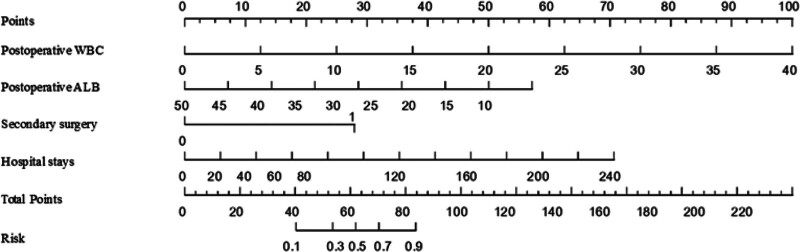
Prediction model for perioperative gram-negative bacteria infection in patients with intestinal fistula.

**Figure 6.**
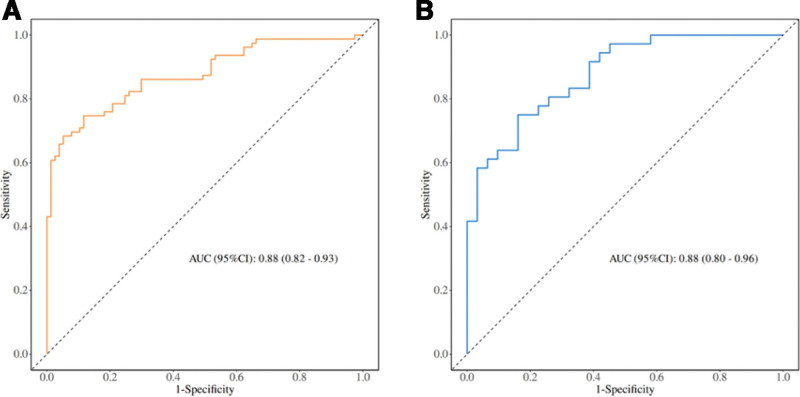
Performance evaluation of nomograph using receiver operating characteristic (ROC) curve. (A) ROC curve of the training set. (B) ROC curve of the validation set.

Based on the variables determined by logistic regression analysis, we constructed a nomogram to intuitively represent the prediction model. This model estimates the probability of perioperative gram-negative bacteria infection in patients with intestinal fistula, as shown in Figure 5. For each predictive variable incorporated into the model, identify the patient’s specific value on the corresponding variable axis, project it vertically upwards onto the score axis to obtain the individual score for that particular variable. Summing the individual scores of all variables yields the total score. Subsequently, locate the calculated total score on the total score axis, project it vertically downwards onto the predictive probability axis, and the corresponding value represents the probability of perioperative gram-negative bacterial infection for the patient in question.And we drew the ROC curve, as shown in Figure 6. In the training set, the area under the curve (AUC) was 0.88 (95% confidence interval [CI], 0.82–0.93) (Fig. 6A). In the validation set, the AUC was also 0.88 (95% CI, 0.80–0.96) (Fig. 6B).

### 3.7. Calibration ability of predictive models

**Figure 7.**
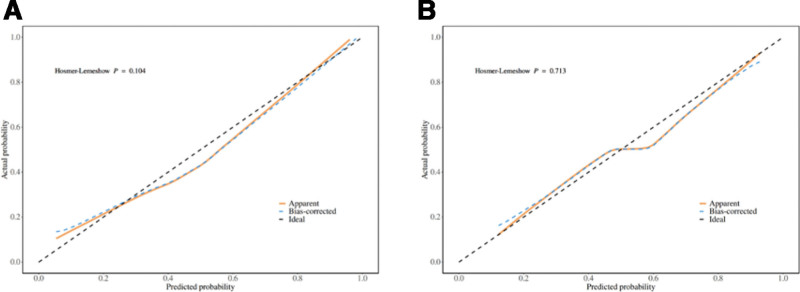
Goodness of fit for prediction of perioperative gram-negative bacteria infection in patients with intestinal fistula. (A) Calibration curve of the training set. (B) Calibration curve of the validation set.

The calibration curve shown in Figure 7 shows that our model prediction is highly consistent with the actual observations of the training set and the validation set. Through Hosmer–Lemeshow goodness of fit test, the regression model trained on the training set showed robust performance (*P* = .104) (Fig. 7A). In addition, a non-significant *P*-value of .713 was also generated in the validation set (Fig. 7B), which confirmed the stability of the model.

### 3.8. Clinical application of prediction models

**Figure 8.**
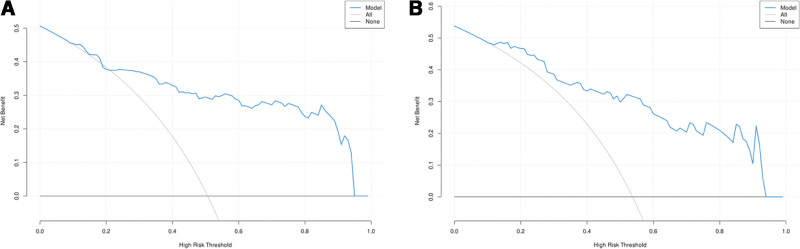
DCA curve of the risk prediction model of perioperative infection with gram- negative bacteria in patients with intestinal fistula. (A) DCA curve of the training set. (B) DCA curve of the validation set. DCA = decision curve analysis.

Figure 8 shows the decision curve analysis of the prediction model. The standardized net income is represented by the y-axis, and the risk threshold probability is represented by the x-axis. The net income related to the forecast model is represented by a solid blue line. The black horizontal solid line corresponds to the scenario where it is assumed that no patients have a gram-negative bacterial infection, while the gray solid line reflects the assumption that all patients are infected with gram-negative bacteria. The larger the gap between the blue curve and these 2 lines, the greater the net benefit. Clearly, this prediction model demonstrates considerable clinical utility.

## 4. Discussion

Intestinal fistula ranks among the prevalent and grave complications that may arise after abdominal surgeries or traumas. When it occurs, it can trigger a host of severe issues, including abdominal infections, hemorrhages, electrolyte disturbances, nutritional deficiencies, and dysfunction of multiple organs.^[10]^ Before the 1970s, the mortality rate of patients with intestinal fistula was as high as 50 to 60%. Despite the development of the times, there is still a mortality rate of 15 to 20% among patients with intestinal fistula.^[3]^ The treatment principle for intestinal fistula has shifted from emergency surgical repair to initial drainage, followed by definitive surgery once the infection and inflammation have subsided and the nutritional status has improved.^[3]^ Academician Li created the concept of intestinal fistula damage control and phased treatment strategy. He also pioneered various specialized techniques and methods for treating intestinal fistula, including Li’s double cannula negative pressure flushing and drainage, silicone plugs, intestinal fluid collection and reinfusion, as well as the use of 3D-printed intestinal fistula stents for intestinal cavity isolation, which benefited many patients.^[11]^ Current research indicates that patients with intestinal fistula frequently suffer from severe infections. On one hand, intestinal fluid leakage can cause abdominal infections, retroperitoneal infections, and necrotizing fasciitis of the abdominal wall,^[12]^ among others. On the other hand, due to long-term fasting or parenteral nutrition, patients with intestinal fistula experience damage to the intestinal mucosal barrier function, increased intestinal permeability, and intestinal bacterial translocation, leading to enterogenous infections.^[13]^ Additionally, due to malnutrition and compromised immune function, these patients are more susceptible to infection. If the infection is not handled properly, it will eventually lead to complex abdominal infection and sepsis, and the mortality can reach 20 to 60%.^[14]^ Although the application of many new technologies has greatly improved the success rate of conservative treatment for patients with intestinal fistula, surgery remains a crucial treatment option. Given the frequent comorbidities, complex disease courses, significant individual differences, and varying locations and numbers of fistulas in patients with intestinal fistula, each individual undergoes distinct surgical procedures and timings.^[15]^ This complexity also increases the risk of surgical complications, especially infection. This study is the first to explore the risk factors of perioperative gram-negative bacteria infection in patients with intestinal fistula, in order to establish a predictive model to help clinicians effectively assess the risk of gram-negative bacteria infection in patients who need surgery. In addition, the analysis of bacterial distribution and drug resistance provides a reference for clinical selection of antibiotics.

This single-center, retrospective cohort investigation revealed that among gram-negative bacterial infections in intestinal fistula patients during the perioperative period, *K pneumoniae* was the most common pathogen at 24.48%, followed by *E coli* at 21.35%, *P aeruginosa* at 16.67%, and *A baumannii* at 16.67%. The prominence of *K pneumoniae* as the leading gram-negative bacteria in such infections highlights its significance in clinical settings. A key finding was that the majority of the bacteria identified were multidrug-resistant (91.15%), which we posit is intricately linked to the patients’ clinical profiles and medical histories. Most patients in this study were referrals from other hospitals, having previously undergone 1 or more surgeries and been exposed to a range of antibiotics over extended periods for infection control. As a result, the infection and multidrug resistance rates for these patients during their subsequent treatment at our hospital were markedly elevated compared to those of typical patients. Consequently, multidrug-resistant bacteria, with *K pneumoniae* being predominant, account for the lion’s share of perioperative gram-negative bacterial infections in intestinal fistula patients, necessitating heightened vigilance. This conclusion is also supported by the research of Liu et al.^[16]^

In recent years, the development of risk prediction models has emerged as a focal point in clinical research. However, there are currently limited studies reporting on infection risk prediction models specifically for patients with intestinal fistula. Taking into account the aforementioned influencing factors, we have developed a nomogram prediction model to assess the risk of perioperative gram-negative bacteria infection in patients with intestinal fistula. This model, presented as a visual graph, enhances readability and offers personalized predictions for patients. Our research is founded on an analysis of a dataset comprising 223 patients with intestinal fistula who underwent surgical treatment. Notably, 51.57% of these patients experienced gram-negative bacteria infection during the perioperative period. We identified multiple independent risk factors for perioperative gram-negative bacteria infection in patients with intestinal fistula, including undergoing secondary surgery during hospitalization, prolonged hospital stays and elevated postoperative WBC counts. Notably, a high postoperative ALB level emerged as an independent protective factor against infection. Based on these findings, we developed a risk prediction model. The model’s predictive power and clinical benefits were validated through the plotting of nomograms, ROC curves, calibration curves, and decision curves.

Patients with intestinal fistula face complex conditions and numerous postoperative complications, posing risks of adverse events such as gastrointestinal bleeding and recurrent abdominal infections after surgical treatment. In the event of such postoperative adverse events, surgical intervention is necessary. However, secondary surgery itself imposes a significant burden on patients. The accumulation of surgical trauma further exacerbates systemic inflammatory response, disrupts the local tissue repair environment, and significantly increases the risks of deep incision infection, abdominal abscess formation, and sepsis. Additionally, repeated surgical procedures may also lead to long-term complications such as intestinal adhesion, blood supply disorders, and even short bowel syndrome. A study involving 90,263 patients undergoing pulmonary surgery also confirmed that patients undergoing secondary surgery have a higher mortality rate and more postoperative complications.^[17]^ Compared to ordinary patients, patients with intestinal fistula face higher surgical risks, require more complex preoperative preparations, and often result in longer hospital stays. A study has shown that prolonged preoperative hospitalization is directly associated with an increased risk of postoperative hospital-acquired infections. The study found that for every day of preoperative hospitalization, the risk of postoperative hospital-acquired infections increases by 1.38 times.^[18]^ Additionally, a large-scale population-based cohort study revealed that patients with bloodstream infections caused by extended-spectrum beta-lactamase-producing Escherichia coli had hospital stays that were 14% longer than those of non-infected patients.^[19]^ White blood cells (WBC), the most commonly utilized inflammatory biomarker in clinical practice, typically serve as indicators of infection when elevated.^[20]^ Intestinal fistulas can result in persistent contamination of the peritoneal cavity with bacteria-laden intestinal contents, precipitating localized infections (e.g., intra-abdominal abscesses) and triggering a systemic inflammatory response. This process stimulates the release of a substantial number of neutrophils from the bone marrow, leading to a marked increase in WBC count. Consequently, elevated WBC levels represent a compensatory mechanism employed by the body to combat infection. Albumin, the predominant protein in plasma, not only contributes to the maintenance of plasma osmotic pressure but also plays a pivotal role in immune regulation and the inflammatory response. It binds to and neutralizes pathogenic factors such as endotoxins, thereby attenuating the cascade amplification effect of systemic inflammatory response syndrome (SIRS).^[21]^ Simultaneously, ALB functions as a primary antioxidant in the body, with its molecular sulfhydryl groups effectively scavenging the copious oxygen free radicals generated during infection and stress. This action mitigates damage to cell membranes, proteins, and DNA, thereby preserving the functional integrity of immune cells.^[22]^ Recent studies have also indicated that fluctuations in ALB levels can serve as valuable biomarkers for assessing infection severity and predicting infection outcomes.^[23,24]^

In this study, we focused on the intraoperative blood loss and the number of double-lumen cannulas in patients with intestinal fistula. The findings revealed that both intraoperative blood loss and the number of double-lumen cannulas were notably higher in the infection cohort compared to the non-infection cohort. On one hand, increased intraoperative blood loss may necessitate more blood transfusions. Research indicates that the risk of infection escalates by 7.6% with each additional unit of blood transfusion.^[25]^ On the other hand, intraoperative blood loss results in the depletion of circulating serum antibiotics, which, through volume resuscitation and blood transfusion dilution, impacts antibiotic concentration, thereby elevating the risk of infection.^[26,27]^ While the utilization of double-lumen cannulas for negative pressure irrigation and drainage post-surgery in intestinal fistula patients can significantly minimize the risk of abdominal infection, we posit that excessive use of double-lumen cannulas could significantly heighten the likelihood of external pathogen invasion. This underscores the importance of rationalizing the use of double-lumen cannulas.

This study holds academic significance and demonstrates innovation in the realm of infection prediction following intestinal fistula surgery. When compared to previously documented prediction models, this research exhibits notable disparities in terms of outcome definition, population focus, and variable selection. For instance, the GloSSI model, grounded in a global multicenter gastrointestinal surgery cohort (comprising a training set of 14,019 cases with an AUC of 0.738), integrates 6 variables: national income level, ASA classification, diabetes, surgical contamination degree, surgical approach, and surgical duration—to forecast surgical site infection (SSI) within 30 days post-surgery.^[28]^ The model’s strength lies in its generalizability and validation through a large sample size; however, it does not differentiate between pathogen types in its outcome definition and encompasses all gastrointestinal surgeries without specifically addressing the unique subpopulation of intestinal fistulas. Another retrospective study (n = 264) identified preoperative non-thyroid illness syndrome (NTIS) and multiple intestinal fistulas as independent predictors of postoperative SSI in patients with external intestinal fistulas.^[29]^ This study pioneered the incorporation of thyroid function status into intestinal fistula infection prediction but similarly did not stratify infectious pathogens by type.

In contrast, this study concentrates its predictive efforts on “perioperative gram-negative bacterial infections” rather than broadly defined surgical site infections (SSIs) or abdominal infections. Given the severe abdominal contamination experienced by patients with intestinal fistulas, with gram-negative bacteria being the predominant pathogens, utilizing pathogen-specific indicators as outcome variables enhances the predictive model’s clinical applicability in guiding antimicrobial therapy selection. Moreover, the variables included in this study comprehensively reflect the distinctive pathophysiological characteristics of intestinal fistula patients, aligning more closely with clinical specialty practices.

Regarding clinical application, the nomogram developed in this study offers clear bedside operability and valuable decision-making guidance. Firstly, it facilitates preoperative risk stratification and enables individualized adjustments to antimicrobial prophylaxis strategies: for patients scoring in the upper quartile range of the nomogram (e.g., with a predicted probability exceeding 60%), enhancing the empirical antimicrobial regimen to cover *P aeruginosa* or extended-spectrum beta-lactamase-producing Enterobacteriaceae, in addition to standard prophylaxis, may be considered; for extremely high-risk patients with a predicted probability over 80%, formulating a preoperative anti-infection plan in collaboration with the surgical critical care team in advance is advisable. Secondly, the model aids in guiding postoperative resource allocation: high-risk patients may benefit from increased frequency of microbial cultures of drainage fluid and extended indwelling times for double-lumen cannulas, among other measures.

Nevertheless, this study is not devoid of limitations. Firstly, as a single-center retrospective study, it may be susceptible to inherent biases. Additionally, the sample size is relatively constrained, precluding the construction of sub-models or interaction tests for subgroups such as high small intestinal fistulas, colonic fistulas, and multiple fistulas. In practical settings, the model’s predicted probabilities may exhibit a tendency towards underestimation. Therefore, clinicians should exercise comprehensive judgment, taking into account specific patient conditions, fistula location, number of fistulas, and daily intestinal fluid loss. Thirdly, owing to the intricate disease course in intestinal fistula patients, many of whom seek treatment at multiple hospitals, and the statistical challenges posed by certain treatment modalities (e.g., antibiotic use and enteral/parenteral nutrition), these factors were not incorporated into the study. To address these limitations, future research endeavors should prioritize multi-center prospective investigations to enhance the representativeness and diversity of patient cohorts, and undertake stratified modeling for various intestinal fistula subtypes to further augment the model’s applicability to complex intestinal fistula populations.

## 5. Summary

This research examined the prevalence of gram-negative bacteria in patients with intestinal fistula during the perioperative period and revealed that these bacteria are primarily multidrug-resistant, with *K pneumoniae* being the most common. The study identified secondary surgery during hospitalization, extended hospital stays, and elevated postoperative WBC counts as independent risk factors for infection. Conversely, a higher postoperative ALB level was found to be a protective factor. Using these indicators, we constructed and validated a reliable predictive model that serves as a practical tool for clinicians. This model could facilitate the rational use of antibiotics and improve outcomes for patients with intestinal fistula.

## Author contributions

**Conceptualization:** Yong-Hao Sun.

**Data curation:** Yong-Hao Sun, Kun-Jian Wei, Ming-Ming Yin, Hao-Yi Zheng.

**Formal analysis:** Yong-Hao Sun, Kun-Jian Wei, Ming-Ming Yin, Hao-Yi Zheng.

**Funding acquisition:** Can-Wen Chen, Guo-Sheng Gu.

**Investigation:** Kun-Jian Wei.

**Methodology:** Yong-Hao Sun.

**Project administration:** Guo-Sheng Gu.

**Resources:** Can-Wen Chen, Guo-Sheng Gu.

**Supervision:** Guo-Sheng Gu.

**Validation:** Hao-Yi Zheng, Qun Xu, Yi-Qun Shan.

**Visualization:** Kun-Jian Wei, Qun Xu, Yi-Qun Shan, Ying-Nan Tian.

**Writing – original draft:** Yong-Hao Sun.

**Writing – review & editing:** Yong-Hao Sun, Can-Wen Chen.
